# A High Diversity in Chitinolytic and Chitosanolytic Species and Enzymes and Their Oligomeric Products Exist in Soil with a History of Chitin and Chitosan Exposure

**DOI:** 10.1155/2015/857639

**Published:** 2015-07-26

**Authors:** Malathi Nampally, M. B. Govinda Rajulu, Dominique Gillet, T. S. Suryanarayanan, Bruno B. Moerschbacher

**Affiliations:** ^1^Institute for Biology and Biotechnology of Plants, WWU Münster, Schlossplatz 8, 48143 Münster, Germany; ^2^Research and Development Laboratory, Sri Biotech Laboratories India Ltd., Hyderabad 500 034, India; ^3^Vivekananda Institute of Tropical Mycology (VINSTROM), Ramakrishna Mission Vidyapith, Chennai 600 004, India; ^4^Gillet Chitosan EURL, Laurent Bonnevay 17, 54100 Nancy, France

## Abstract

Chitin is one of the most abundant biomolecules on earth, and its partially de-N-acetylated counterpart, chitosan, is one of the most promising biotechnological resources due to its diversity in structure and function. Recently, chitin and chitosan modifying enzymes (CCMEs) have gained increasing interest as tools to engineer chitosans with specific functions and reliable performance in biotechnological and biomedical applications. In a search for novel CCME, we isolated chitinolytic and chitosanolytic microorganisms from soils with more than ten-years history of chitin and chitosan exposure and screened them for chitinase and chitosanase isoenzymes as well as for their patterns of oligomeric products by incubating their secretomes with chitosan polymers. Of the 60 bacterial strains isolated, only eight were chitinolytic and/or chitosanolytic, while 20 out of 25 fungal isolates were chitinolytic and/or chitosanolytic. The bacterial isolates produced rather similar patterns of chitinolytic and chitosanolytic enzymes, while the fungal isolates produced a much broader range of different isoenzymes. Furthermore, diverse mixtures of oligosaccharides were formed when chitosan polymers were incubated with the secretomes of select fungal species. Our study indicates that soils with a history of chitin and chitosan exposure are a good source of novel CCME for chitosan bioengineering.

## 1. Introduction

Shrimp and crab shell wastes are used commercially for the extraction of chitin which can then be converted into its partially de-N-acetylated forms, chitosans. Chitosans are a family of molecules differing with respect to their degree of polymerisation (DP), degree of acetylation (DA), and pattern of acetylation (PA). Such variations influence the physicochemical solution properties as well as the biological functionalities of chitosans [1–5] which find use in agricultural, food, and pharmaceutical industries [6]. Therefore, well characterized chitosans with broad ranges of specific DPs, DAs, and PAs are crucial for detailed structure-function analyses. To this end, chitin and chitosan modifying enzymes (CCMEs) such as chitinase, chitin deacetylase, and chitosanase could be used to complement the chemical methods currently used for this purpose [7–9]. Furthermore, chitosans could be broken down to soluble derivatives called chitooligosaccharides (CHOS) which are vested with desirable technological properties [10]. Since enzymatic conversion of chitin to chitosan and CHOS is ecofriendly, more specific and a cheaper option compared to the chemical methods [11] and could potentially augment the existing chemical methods [12, 13] for characterization of chitosans, search for novel CCME is a worthwhile exercise. With the expectation that soils with a long history of exposure to chitin and chitosan would select organisms elaborating diverse chitinolytic and chitosanolytic enzymes, we proceeded with the current work.

Although fungi are reported to contribute more than bacteria to environmental degradation of chitin [14], much less is known about the fungi involved compared to chitinolytic bacteria; bacterial CCMEs have been studied in much more detail than fungal enzymes. In terrestrial soils, the most prevalent chitin degrading bacteria are species of* Bacillus, Stenotrophomonas,* Gammaproteobacteria, and* Arthrobacter* [15, 16] while those in marine sludges are species of* Actinobacterium, Pantoea,* and* Pseudomonas* [17, 18]. Fungi such as* Trichoderma viride* [15] and species of* Mortierella* and* Fusarium* isolated from soil exhibit appreciable chitinolytic activity in the presence of chitin in the culture medium [19]. Here, we looked at the diversity of chitinolytic and chitosanolytic fungi and bacteria in soils of a chitin and chitosan producing company in Gujarat, India. These soils had been exposed to dry or fresh shrimp shells or to chitin or different types of chitosan for more than ten years. In addition to species diversity, we also analysed the diversity of CCME present in these organisms, as well as the diversity of products produced by these CCMEs.

## 2. Materials and Methods

### 2.1. Soil Samples

Seven soil samples were collected from different sites of Mahtani Chitosan Pvt. Ltd., Veraval (Gujarat, India), a chitin/chitosan producing company from a depth of 5 to 10 cm, and stored at 4°C for a maximum of two months before further processing.

### 2.2. Preparation of Colloidal Chitin and Chitosans

Colloidal chitin was prepared according to the method of Berger and Reynolds [20]. To 10 g of *β*-chitin, 500 mL of conc. HCl was added, stirred to get a homogeneous mixture, and incubated at 4°C overnight. Two litres of double-distilled water was then added, stirred for 48 hours at 4°C, and then washed with double-distilled water until the pH was neutral.

Chitosan (average DA 3%, average DPn ca. 2,000) was dissolved in an aqueous acetic acid (0.1 M) solution and purified by successive filtration and extensive washing by repeated precipitation and centrifugation; following this, chitosans with DA 35%, DP 900, and DA 50%, DP 820 were prepared by partial re-N-acetylation using acetic anhydride in 1,2-propanediol [21]. The DA of the resulting chitosans was determined using ^1^H NMR spectroscopy [22], and the DP using HP-SEC coupled to RI and MALLS detectors [3].

### 2.3. Preparation of Chitin and Chitosan Agar Plates

For visualizing chitinolytic activity, Petri dishes with M9 minimal medium amended with 0.5% of colloidal chitin as sole carbon source were used. Petri dishes with Luria-Bertani (LB) agar medium with 0.9% chitosan (DA 3%) as the sole carbon source were used to identify chitosanolytic activity. The appearance of a clear zone around the colony of a bacterium or fungus growing on M9 and LB medium indicated chitinolytic and chitosanolytic activity, respectively.

### 2.4. Isolation of Chitinolytic and Chitosanolytic Fungi and Bacteria from Soil Samples

Fungi were isolated from the soil samples by dilution plating and Warcup soil-plate methods [23]. For dilution plate, 2 g of soil was suspended in 1 mL of sterile distilled water, and tenfold dilutions of this were spread on PDA (Difco Potato Dextrose Agar medium, Becton and Dickinson, Sparks, USA) plates containing chloramphenicol (150 mg/L) to obtain individual fungal colonies. For soil plates, 2 g of soil was placed in a sterile Petri dish, cooled PDA medium (15 mL) was added, and soil particles were spread in the medium. All plates had replicates and were incubated at 28°C for 20 days to obtain fungal colonies. The fungi were isolated and subcultured in PDA slants. Slide cultures of these isolated fungi were then prepared, stained, and observed under microscope to identify them based on standard keys [24].

Bacteria were isolated using a modified serial dilution method of Maltseva and Oriel [25]. Initially, 10 g of soil was inoculated in M9 minimal medium amended with colloidal chitin (0.5%) to enrich chitinolytic bacteria; a few mL of the enriched cultures was spread on LB agar plates and incubated at 37°C for isolating bacteria. Pure cultures were obtained by restreaking the colonies several times until single colonies were obtained.

### 2.5. Preparation of Samples for Zymography and Thin Layer Chromatography

Each fungal isolate was grown in potato dextrose broth for 5 days as static culture at 28°C and 100 mL of this culture filtrate (secretome) was dialyzed (MWCO 1,000 kDa) for 24 h against distilled water at 4°C; 10 mg of the lyophilized secretome was mixed in 1 mL of 50 mM sodium acetate buffer (pH 5.2) and centrifuged at 16,000 g for 5 min (20°C). An aliquot (5–10 *μ*L) of sample was used for dot activity assay or zymography. Bacterial isolates were grown in 10 mL of LB medium for 48 h at 37°C, centrifuged, and the secretomes were lyophilised. Lyophilised samples were dissolved in 1 mL of 5 mM sodium acetate buffer (pH 5.0) and used for assessing enzyme activities.

### 2.6. Detection of Chitinase and Chitosanase Activity by Dot Assay and Zymography

Chitinolytic and chitosanolytic enzyme activities were detected using a dot activity assay as described previously [26]. Briefly, 5 *μ*L from a fungal secretome preparation was applied on the gels prepared with glycol-chitin (0.3 mg/mL) or chitosan DA 35% (0.1 mg/mL). Gels were incubated at 37°C overnight and then stained l with Calcofluor White. A dark spot under UV transillumination on the gel indicated enzyme activity.

For detecting isoenzymes, seminative SDS-PAGE (12%) was run in gels containing 0.3 mg/mL of glycol-chitin for chitinase or 0.1 mg/mL of either of two chitosans (DA 50% or DA 35%) [27]. After electrophoresis (50 mA for 4 h), the gel was washed twice for 20 min each in 50 mM sodium acetate buffer (pH 5.2, with 1% Triton X-100), followed by two washes in buffer without Triton X-100. The gel was incubated at 37°C for 12 h under shaking in 50 mM sodium acetate buffer (pH 5.2) and stained with 0.01% Calcofluor White (Sigma, Steinheim, Germany) in 0.5 M Tris/HCl-buffer (pH 8.9) for 5 min and finally washed in deionized water for 1 h. The isozymes were visualized on a UV transilluminator. A crude extract of a known chitinolytic strain of* Bacillus licheniformis* [28] was run as a positive control.

Alternatively, zymography was done using isoelectric focusing (IEF) over the pH range 3–10 in a polyacrylamide gel containing Ampholine (Amersham Bioscience, Uppsala, Sweden) followed by activity staining using overlay gels containing 0.1 mg/mL of either of the chitosans mentioned above. After incubation in 50 mM ammonium acetate buffer (pH 5.2) overnight at 37°C, overlay gels were stained with Calcofluor White as described above.

### 2.7. Detection of Chitosan Oligomers by Thin Layer Chromatography

A sample (20 *μ*L) of secretome of selected fungal isolates was mixed with 20 *μ*L of chitosan DA 35% solution (1 mg/mL) and incubated overnight at 37°C in 50 mM sodium acetate buffer (pH 5.5). Samples were concentrated under reduced pressure to scale down the volume, and aliquots of 10 *μ*L were applied on TLC plates (Merck, Berlin, Germany), run in butanol : methanol : ammonia : water (5 : 4 : 2 : 1, v/v/v/v) and stained using aniline-diphenylamine reagent (4 mL of aniline, 4 g of diphenylamine, 200 mL of acetone, and 30 mL of 85% phosphoric acid). Oligomers were visualised by heating the plate at 180°C for 3–5 min. Oligomers were compared with authentic N-acetyl-D-glucosamine (DP 1, 2, 3, 5, 6) and D-glucosamine (DP 1, 3, 4) standards (Seikagaku, Tokyo, Japan).

### 2.8. 16S-rDNA Analysis of Bacterial Isolates

PCR was performed on the bacterial soil isolates using bacterial universal primers: forward primer (5′AGAGTTTGATC(AC)TGGCTCAG3′), reverse primer (5′AAGGAGGTGATCCA(AGCT)CC(AG)CA3′) [29]. Amplicons were cloned into PCRII-TOPO vector and sequenced at MWG, Ebersberg, Germany. Blast analyses were performed with the sequences in the NCBI database. Sequences obtained were deposited in NCBI under Gene Bank with sequence id's JN593073–JN593080.

## 3. Results

### 3.1. Isolation and Screening for Chitinolytic or Chitosanolytic Bacteria and Fungi

**Figure 1:**
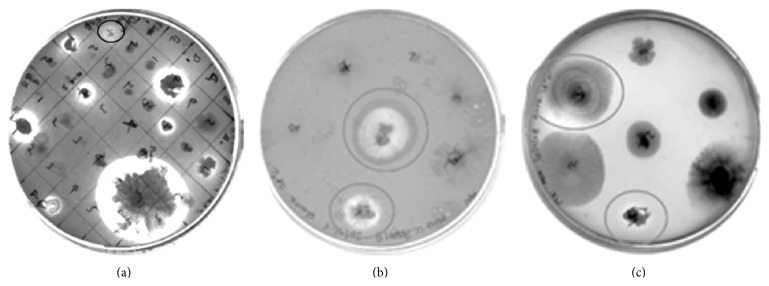
Chitinolytic and chitosanolytic activities in bacterial and fungal isolates from soil samples. (a) Bacterial strains showing clearing zones on minimal medium agar plates containing colloidal chitin; one strain showing weak chitinolytic activity (top, marked with circle) was excluded from further studies as it did not show the activity consistently; (b) and (c) examples of fungal strains showing clearing zones on agar plates containing colloidal chitin in minimal medium (b) or chitosan DA 3% in LB medium (c).

A total of sixty bacterial strains were isolated from seven soil samples collected from different sites of the chitin/chitosan producing company. On a minimal medium with 0.5% colloidal chitin as the sole carbon source, eight strains consistently produced clear zones around their colonies indicating a chitinolytic activity (Figure 1(a)). Microscopic observations indicated that all of them were* Bacillus* species differing in their motility, sporulation, and arrangement of spores. The overall 16S-rDNA sequence identity between these* Bacillus* strains ranged from 99.5 to 100%. 16S-rDNA sequence identities of 99 and 99.7% corroborated this to the* cereus/anthracis/thuringiensis* group of* Bacillus*.

A total of 25 fungal strains were isolated from two different soil samples. Many of them were* Aspergillus* species; other genera included* Acremonium*,* Aureobasidium*,* Cladosporium*,* Curvularia*,* Drechslera*,* Fusarium*,* Penicillium*, and* Sporormiella* (Table 1). Of the seven randomly chosen isolates from these, two produced clearing zones on chitin medium (Figure 1(b)), and two others on chitosan medium (Figure 1(c)). Of the 25 isolates, 13 were positive for chitinase, 14 were positive for chitosanase, and 7 produced both the enzymes as visualized by dot assay (Table 1). Among the 20 fungal isolates which were chitinolytic and/or chitosanolytic, 14 identified fungi belong to seven different genera, namely*, Acremonium, Aspergillus*,* Aureobasidium*,* Cladosporium*,* Drechslera, Fusarium*, and* Penicillium* which are common saprotrophs found in soils [30].

### 3.2. Chitinolytic and Chitosanolytic Enzymes of Bacterial and Fungal Isolates

**Figure 2:**
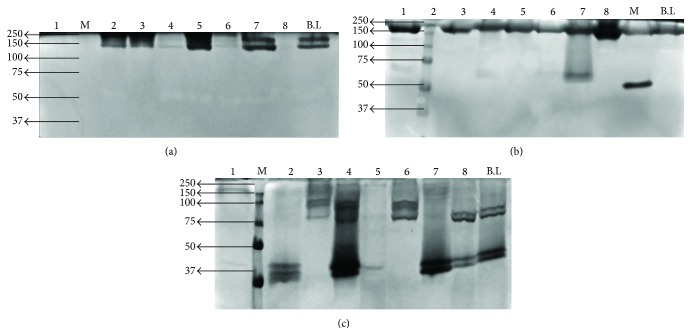
Seminative SDS-PAGE of crude extracts of the bacterial soil isolates (1–8), followed by zymography using glycol-chitin (a), chitosan DA 56% (b), or chitosan DA35% (c) as a substrate. A known chitinolytic strain of* Bacillus licheniformis* (B.L) was used as a positive control. The positions of marker proteins (M) are given on the sides of the gels.

The crude extract of* B. licheniformis* (control) showed activity on all three substrates whereas the secretomes from the different soil bacterial strains showed differences in their activities. Isolates 2, 3, 5, and 7 showed the same two high-molecular weight chitinases as* B. licheniformis,* while the extracts from isolates 1, 4, 6, and 8 were not active on glycol-chitin (Figure 2(a)). All isolates including* B. licheniformis* produced one high MW isoenzyme degrading chitosan (DA 56%) and isolates 2, 4, and possibly 7 possessed an additional isoenzyme with a MW between 50 and 75 kDa capable of degrading this chitosan (Figure 2(b)). All of the strains including* B. licheniformis* had isoenzymes degrading chitosan DA 35% (Figure 2(c)). Isolates 1 and 5 produced fewer and weakly active chitosanase isoforms and isolates 4 and 7 produced chitosanase of highest MW. Considering the activities on all three substrates, it was clear that all eight isolates differ from each other and from* B. licheniformis* in their chitinolytic and chitosanolytic isoenzymes, but their diversity was limited.

PCR was performed on genomic DNA of the eight bacterial soil isolates using primers designed from conserved regions of known* Bacillus* chitosanases. Amplicons were observed at 1.3 Kb only in strains 1, 3, 6, and 7 (data not shown); the other strains did not show any amplification. Blast results showed that the sequences were identical to the known chitosanase sequence of* Bacillus* sp. strain KCTC 0377BP [31].

**Figure 3:**
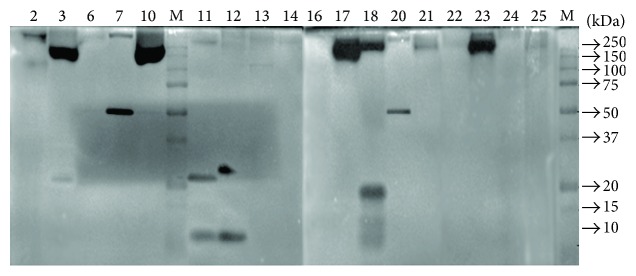
Seminative SDS-PAGE of crude extracts of selected fungal soil isolates (numbers correspond to Table 1), followed by zymography using chitosan DA 35% as a substrate. The positions of marker proteins (M) are given on the right side.

To analyse the chitosanolytic isoenzymes of fungi, crude extracts of fungal isolates which were positive in the dot assay with chitosan DA 35% as a substrate were subjected to seminative SDS-PAGE in a gel containing chitosan DA 35% (Figure 3). Isoenzyme activity was observed in all isolates; the isolates differed in the number of isoforms and in their overall activity. The number of isoenzymes ranged from one to three and their MW ranged from very low to very high. Isolates 3, 10, 17, 18, and 23 (*Penicillium* and all three chitosanolytic* Aspergillus* isolates) had a strong activity at MW of ca. 250 kDa; two of the three* Cladosporium* isolates (7 and 20) showed one sharp band around 50 kDa; isolates 11, 12, and 18 had one or two low MW isoforms between 10 and 20 kDa.

**Figure 4:**
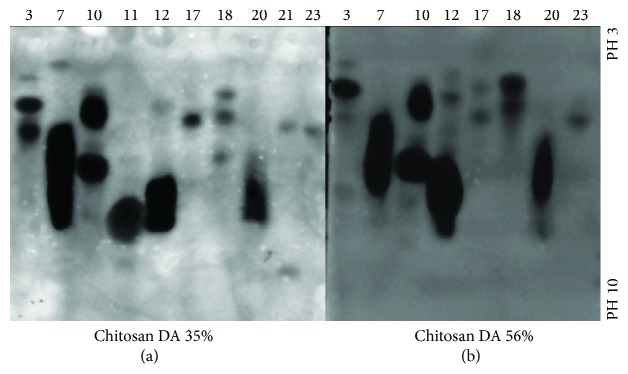
Isoelectric focusing of crude extracts of selected fungal soil isolates (numbers correspond to Table 1), followed by zymography using overlay gels containing chitosan DA 35% (a) or chitosan DA 56% (b) as a substrate. The pH range of the gels is indicated at the right side.

Samples which differed clearly in their isoenzyme spectrum were selected, and their proteins were separated by isoelectric focusing (IEF). For zymography, polyacrylamide gels containing different chitosans with DA 35% and DA 56% were overlaid on the IEF gel after the run (Figure 4). Gels were incubated at 37°C overnight and stained with Calcofluor White to detect chitosanolytic activity. All fungal isolates had one to four chitosanolytic isoenzymes with isoelectric points ranging from pH 4 to pH 8. While few differences were seen between the two substrates tried, clear differences were obvious between the different isolates.

### 3.3. Chitosan Oligomers Produced by Chitosanolytic Enzymes of Select Fungal Isolates

**Figure 5:**
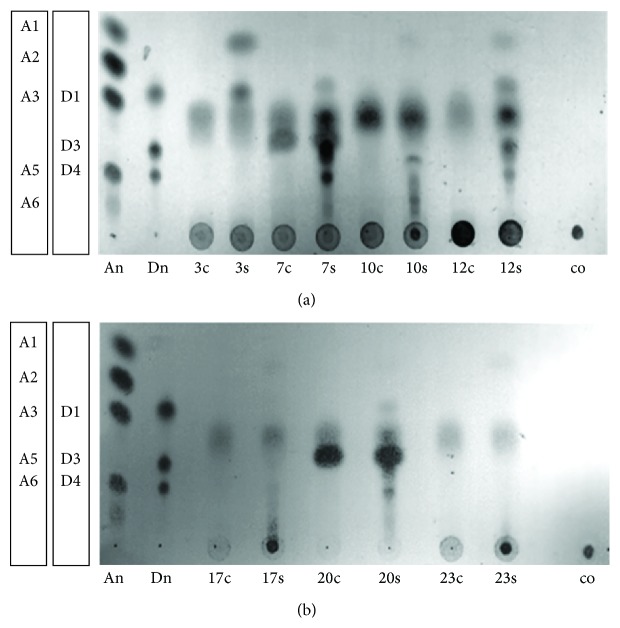
(a) and (b). TLC analysis of the products of chitosan (DA 35%) incubation with crude extracts of selected fungal soil isolates (numbers correspond to Table 1). Crude extracts were incubated with (samples labeled 3s, 7s, 10s, 12s, 17s, 20s, and 23s) or without chitosan (samples labeled 3c, 7c, 10c, 12c, 17c, 20c, and 23c) as a substrate. Chitosan incubated without any crude extract (co) was used as a control, and oligomers of GlcNAc (An) and GlcN (Dn) were used as standards. The DP of the standards is given on the left sides of the plates.

Secretomes from fungal isolates which showed a single dominant isoenzyme in zymography (isolates 3, 7, 10, 12, 17, 20, and 23) were incubated with chitosan DA 35% overnight at 37°C, and the chitosan oligomers produced were analysed using TLC (Figures 5(a) and 5(b)). This preliminary analysis showed that different oligomer mixtures were produced by each fungal isolate, ranging from the monomers GlcN and GlcNAc (isolates 3, 23) to a mixture of small oligomers ranging in degree of polymerization from 2 to 6 (isolates 7, 10, 12, and 20). Isolate 17 produced only larger oligomers.

## 4. Discussion

We argued that soils with a long history of exposure to chitin and chitosan would select microbes with an ability to degrade chitin and/or chitosan. Hence, we studied the soils of a chitin and chitosan producing company which has been processing ca. 5,000 tons of fresh and dried shrimp annually since the year 1995. Earlier, our collaborators from India reported that Gammaproteobacteria were dominant in these soils [16]. We found eight different* Bacillus* species belonging to the* cereus/anthracis/thuringiensis* group which is well known for their potential to degrade chitin and chitosan [32–35]. With regard to the CCME, chitosanases were more diverse than chitinases in these species. Using degenerate primers of known* Bacillus* chitosanases [36], we could amplify a chitosanase gene from four of the eight strains which was identical to a chitosanase gene from* Bacillus* sp. strain KCTC 0377BP [31]. We have now set up a pooled genomic DNA library of these strains and are screening it for chitinase and chitosanase genes.

Although fungi with CCME activities have been reported from soils [37–39], to our knowledge, this is the first report on fungal diversity in soils with a history of chitin/chitosan exposure. We identified the fungi based on spore morphology, but a molecular approach will be essential to authenticate their identity at the species level. Though fungi produce chitinolytic enzymes* per se* for cell wall remodelling during their developmental processes [40], the diversity of these enzymes could be higher in fungi present in soils with spent chitin material since the fungi here could possibly be utilizing chitin and chitosan polysaccharides as carbon and nitrogen source [41]. This could be the reason why almost all of the fungi screened here were positive for chitin and/or chitosan degrading enzymes: 13 were chitinolytic, 14 were chitosanolytic, and 7 isolates were both chitinolytic and chitosanolytic. Furthermore, isolates belonging to the same genus differed significantly in their chitinolytic and chitosanolytic potential. A typical case is the genus* Aspergillus* which dominated the fungal isolates. Of the five* Aspergillus* isolates, one was chitinolytic but not chitosanolytic, one was chitosanolytic but not chitinolytic, two were both chitinolytic and chitosanolytic, and one was neither chitinolytic nor chitosanolytic. Similarly, of the two* Acremonium* isolates, one was negative for both activities and one was positive for both activities; all three* Cladosporium* isolates were chitosanolytic but only one was also chitinolytic; both the* Drechslera* isolates were chitinolytic and one was also chitosanolytic. Thus, soils with a history of chitin and chitin exposure appear to be a promising source of fungi with high CCME diversity useful for technological exploitation. This assumption was further substantiated by the isoenzyme patterns of chitosanases discerned based on size and isoelectric point. Most fungal isolates produced more than one chitosanolytic isoenzyme. IEF was superior to native PAGE for visualizing isozymes as more isoforms were visible for isolates 3, 10, 12, 17, and 20 in the latter method of detection. A few fungal isolates which tested positive for chitosanase in the dot activity assay (with chitosan DA 35% as substrate) showed weak activities in the zymograms with this substrate possibly owing to improper renaturation of the enzymes when the SDS was washed out. In spite of the multiplicity of enzymes, chitosan DA 35% was not fully degraded to monomers or very small oligomers by any of the crude enzyme preparations; pentamers and larger oligomers were produced by isolates 7, 10, 12, 17, and 20.

## 5. Conclusions

In conclusion, we observed strong and diverse chitinolytic and chitosanolytic activities among the microorganisms present in soil samples with a history of chitin/chitosan exposure, and the diversity in fungal species and their CCME here was higher than the bacterial diversity. Analysing the biodiversity of microorganisms in an environmental sample by screening for the diversity of isoenzymes and for the oligomers produced by these enzymes is a novel but promising approach. The high diversity found is of biotechnological relevance as isolated bacterial and fungal chitinases and chitosanases [42, 43] as well as the oligomers produced by purified or crude chitinolytic and chitosanolytic enzymes [31, 44, 45] have interesting and diverse biological activities and may, thus, be useful in a wide range of applications.

## Figures and Tables

**Table 1:** Chitinase and chitosanase activity of the fungal isolates screened from soil samples.

Isolate number	Name of the fungus	Activity in dot assays	
		Chitinase^∗^	Chitosanase^∗^
1	*Fusarium* sp.	+	−
2	Unidentified	−	+
3	*Penicillium* sp.	+	+
4	*Aspergillus* sp.	+	−
5	*Acremonium* sp.	−	−
6	*Cladosporium* sp.	+	+
7	*Cladosporium* sp.	−	+
8	*Aureobasidium pullulans *	+	−
9	*Aureobasidium pullulans *	+	−
10	Unidentified	+	+
11	Unidentified	−	+
12	Unidentified	−	+
13	Unidentified	+	−
14	*Aspergillus* sp.	−	−
15	*Curvularia* sp.	−	−
16	Unidentified	−	−
17	*Aspergillus* sp.	+	+
18	*Aspergillus niger *	+	+
19	*Sporormiella intermedia *	−	−
20	*Cladosporium cladosporioides *	−	+
21	*Drechslera* sp.	+	+
22	*Drechslera* sp.	+	−
23	*Aspergillus* sp.	−	+
24	*Acremonium* sp.	+	+
25	Unidentified	−	+

^∗^Glycol-chitin and chitosan DA 36% were used as substrates to detect chitinase and chitosanase activity, respectively; + = positive; − = negative.
